# Temperature-dependent phenology of *Plutella xylostella* (Lepidoptera: Plutellidae): Simulation and visualization of current and future distributions along the Eastern Afromontane

**DOI:** 10.1371/journal.pone.0173590

**Published:** 2017-03-16

**Authors:** Benignus V. Ngowi, Henri E. Z. Tonnang, Evans M. Mwangi, Tino Johansson, Janet Ambale, Paul N. Ndegwa, Sevgan Subramanian

**Affiliations:** 1 International Centre of Insect Physiology and Ecology, Nairobi, Kenya; 2 School of Biological Sciences, University of Nairobi, Nairobi, Kenya; 3 National Plant Quarantine Station, Tropical Pesticides Research Institute, Arusha, Tanzania; 4 International Maize and Wheat Improvement Centre (CIMMYT), Nairobi, Kenya; 5 Department of Geosciences and Geography, University of Helsinki, Helsinki, Finland; USDA Agricultural Research Service, UNITED STATES

## Abstract

There is a scarcity of laboratory and field-based results showing the movement of the diamondback moth (DBM) *Plutella xylostella* (L.) across a spatial scale. We studied the population growth of the diamondback moth (DBM) *Plutella xylostella* (L.) under six constant temperatures, to understand and predict population changes along altitudinal gradients and under climate change scenarios. Non-linear functions were fitted to continuously model DBM development, mortality, longevity and oviposition. We compiled the best-fitted functions for each life stage to yield a phenology model, which we stochastically simulated to estimate the life table parameters. Three temperature-dependent indices (establishment, generation and activity) were derived from a logistic population growth model and then coupled to collected current (2013) and downscaled temperature data from AFRICLIM (2055) for geospatial mapping. To measure and predict the impacts of temperature change on the pest’s biology, we mapped the indices along the altitudinal gradients of Mt. Kilimanjaro (Tanzania) and Taita Hills (Kenya) and assessed the differences between 2013 and 2055 climate scenarios. The optimal temperatures for development of DBM were 32.5, 33.5 and 33°C for eggs, larvae and pupae, respectively. Mortality rates increased due to extreme temperatures to 53.3, 70.0 and 52.4% for egg, larvae and pupae, respectively. The net reproduction rate reached a peak of 87.4 female offspring/female/generation at 20°C. Spatial simulations indicated that survival and establishment of DBM increased with a decrease in temperature, from low to high altitude. However, we observed a higher number of DBM generations at low altitude. The model predicted DBM population growth reduction in the low and medium altitudes by 2055. At higher altitude, it predicted an increase in the level of suitability for establishment with a decrease in the number of generations per year. If climate change occurs as per the selected scenario, DBM infestation may reduce in the selected region. The study highlights the need to validate these predictions with other interacting factors such as cropping practices, host plants and natural enemies.

## Introduction

Diamondback moth (DBM), *Plutella xylostella* (L.) (Lepidoptera: Plutellidae) is a major pest of cruciferous crops [1]. The pest is estimated to cause a global annual yield loss valued at US$ 1.3 billion with control costs of US$ 1.4 billion [2]. In Kenya, Macharia et al [3] reported that DBM contributes to 31% yield loss in farmer-managed cabbage (*Brassica oleraceae* var. *capitata* L.) crop. Success of DBM as a major pest is attributed to its ability to survive under a wide temperature range, prolific reproductive capacity, ability to feed on diverse host plants, and insecticide resistance [4–5].

Numerous studies on population dynamics of DBM have been conducted in the field [6–10] and in the laboratory [7–8,11–15]. Central to these studies is the role of temperature in influencing the life cycle of DBM. Based on accumulated day-degrees, numerous forecasts have been employed to predict the pest dynamics [16]. However, day-degree forecasts can only predict the sporadic occurrence of pests but not the spread [17]. Studies have also established that DBM migrate long ranges [18] and annually invade from regions where they can overwinter [1,11].

A recent study shows that, worldwide, a synergy of laboratory and field-based findings to map DBM activity across a spatial scale is limited [19]. Several distributions have been generated based on the presence data of DBM in a country/region, its seasonal phenology [19] and persistence [20–21]. Using a bioclimatic model created using CLIMEX, Zalucki and Furlong [19] synthesized the core and seasonal distribution map of DBM across the globe. But, there is a need to understand and predict likely changes in DBM population dynamics in precise hotspot regions, to adopt location-specific and effective management strategies. Moreover, the CLIMEX-based approach mainly fails to consider the vulnerabilities of discrete stages of DBM to extreme temperatures and other biological characteristics of species [22]. In other instances, the forecast was developed using a Monte Carlo simulation from a fixed number of individuals between successive generations rather than targeting the whole population [17].

Built under the physiological timeline of development, phenology models employed in this work are reliable at predicting population dynamics of insect pests [23]. This modeling approach was applied to predict a decrease in the development times of eggs of *Busseola fusca* from 19.1 to 6.5 days between 15 and 30°C, respectively [24–26]. Phenology models are process-oriented mathematical expressions that link climate variability with lesser levels of abstraction to physiological pattern of the insect species growth to mimic and predict the dynamics of their population within a given point location or region [14,27–32].

In East Africa, cruciferous vegetables are grown under ecological conditions from semi-arid low to mid altitude regions to highlands [33]. The Eastern Afromontane transects are characterized with such diverse ecological conditions, and DBM is a key pest of crucifers across the altitude range. Montane ecosystems are vulnerable to climate change and are warming at a greater rate than low elevations [34–35]. Such rapid effects of climate change are likely to influence the physiology and phenology of key insect pests such as DBM, thereby affecting their ability to infest and damage their hosts along the altitudinal gradients. Understanding such changes in the pest’s dynamics is essential, to develop and adopt new pest management strategies. In this regard, the present study focused on the development of temperature-driven phenology models of DBM. Furthermore, the study linked the phenology model with field temperature datasets to estimate the pest demographic parameters and analyze the possible impacts of changes that can occur in the population of DBM along altitudinal transects in the Eastern Afromontane Biodiversity Hotspot (EABH). The phenology model was also mapped at high resolution using downscaled regional climate datasets (AFRICLIM) [36] to predict the changes in the distribution and abundance of DBM along altitudinal transects.

## Materials and methods

### Ethical statement

This study was carried out in cultivated farm plots where all plant species used (namely common cabbage *Brassica oleraceae* var. *capitata*, kale *Brassica oleraceae* var. *acephala* and Ethiopian mustard *Brassica carinata*) and the insect pest (DBM), are neither endangered nor protected.

The Regional Administrative Secretary of Kilimanjaro (reference letter FA/191/228/01/61) and the Kenya Forest Service (reference letter RESEA/1/KFS/5) granted permission to CHIESA project to conduct the research in Mt. Kilimanjaro and Taita hills, respectively. Individual small-scale farmers owning crucifer farm plots granted CHIESA project the permission to conduct research in their farm plots.

### Study sites

The altitudinal transects considered for the study were located within the Eastern Afromontane Biodiversity Hotspot (EABH) in Taita hills, Kenya and Mt. Kilimanjaro, Tanzania, as detailed in Mwalusepo et al [37]. These transects are ‘hotspots’ for cultivation of cruciferous vegetables throughout the year and DBM is a key constraint. A total of 13 crucifer farms were selected in the Taita hills transect, where the altitude increases from Majengo (830 masl) to Mbangang’ombe farm (1785 masl). The lowland Taita hills area is characterized by grassy fields and small thickets of shrubs and woodlands, which become denser with rising altitude. People have cleared nearly all the natural forest in the extensive undulating mountains for agriculture and human settlements [38]. A similar number of crucifer farms was selected in Mt. Kilimanjaro transect, where the altitude increases from Kisangesangeni B (716 masl) to Marua A farm (1692 masl). This transect is characterized by open fields and small stands of bushland areas in the lowlands. Midway up the transect is a transition between fragmented bushland and “Chagga homegardens”, an agroforestry cropping system vertically characterized by a close intermix of assorted food crops and fodder herbs (≤ 1 m.), coffee (1–2.5 m.), bananas (2.5–5 m.), fuel/fodder trees (5–20 m.) and timber/multipurpose trees (20 ≥ 30 m.) [39]. Sustainably maintained through use of traditional furrow irrigation and recycling of nutrients through farmyard manure, the homegardens are well established within the ecosystem in the higher altitudes. Geographic coordinates and elevations of the sampled farms were recorded using a handheld Global Positioning System (GPS) receiver (Garmin eTrex 30, Garmin International Inc., Taipei, Taiwan). Selected farms in both transects were classified into three designated altitudinal zones (low: 700–1200 metres above sea level [masl]; medium: 1201–1600 masl; high: > 1600 masl). In each selected farm, a data logger (iButton, Maxim Integrated Products Inc., California, USA) was installed and daily mean minimum and maximum temperature recorded from January to December 2013.

### Temperature-dependent life tables for diamondback moth

The DBM population utilized for the establishment of life tables was obtained from crucifer vegetables grown in Taita hills transect. The DBM colony was maintained on common cabbage plants (Gloria, F1 hybrid) at the International Centre of Insect Physiology and Ecology (*icipe*) in Nairobi, Kenya as described by Kahuthia-Gathu et al. [40]. The DBM were raised for one generation to enable the field stock to acclimatize to laboratory conditions.

Freshly laid eggs from the colony were inoculated on individual cabbage leaf bits and placed in glass vials (2.5 cm diameter, 7.5 cm high), lined with absorbent paper towels and covered with a fine mesh sieve ventilated lid. The vials were placed in an incubator (Sanyo MIR– 554; Sanyo Electric Co. Ltd, Japan) at five constant temperatures (10, 15, 20, 25 and 30, each ± 1°C). At 35°C, each egg was inoculated on whole young leaf to delay dehydration, with the stipule end wrapped in wet cotton wool and placed inside a ventilated plastic container (12 x 10.2 x 6.5cm) covered with muslin cloth. All experiments were carried out at 70 ± 10% relative humidity and a 12:12 h (L: D) photoperiod. Egg development was recorded daily until hatching. Eggs that failed to hatch by the end of the experiment were assumed to have died. Freshly cut leaves of cabbage were provided to the larvae after every 1 or 2 days. Development of the larvae and pupae was checked and recorded daily. Developmental time and mortality rates were recorded for egg, larvae and pupae until adult emergence.

To record oviposition data, emerged adults were paired at a 1:1 ratio, placed inside ventilated plastic containers (12 x 10.2 x 6.5cm) and fed with 6% sugar solution absorbed in cotton wool balls. The sugar solution was replaced after every 1–2 days. A dried aluminum foil smeared with cabbage leaf extract was hung inside each container as an oviposition substrate. The oviposition substrate was replaced at 24-hour intervals and the numbers of eggs laid recorded. The total life period of each individual adult maintained in different temperature treatments was recorded.

### Phenology model building and validation using Insect Life Cycle Modeling (ILCYM version 3.0)

The Insect Life Cycle Modeling (ILCYM version 3.0) software [41] developed by the International Potato Centre, Lima, Peru [29] was used to generate temperature-dependent phenology models. ILCYM is an open-source computer-aided tool built on R codes and Java interface, equipped with modules for building process based and temperature dependent phenology models for insect populations. The ‘model builder’ module was used to develop the phenology models whereas the ‘validation and simulation’ module of ILCYM was applied to estimate six demographic parameters—[intrinsic rate of natural increase (r_m_), net reproduction rate (Ro), gross reproduction rate (GRR), mean generation time (GT), finite rate of increase (λ) and doubling time (Dt)] of the species]. Practically, ILCYM inputs experimental life table data to estimate functions for the species development time, development rate, mortality, senescence and fecundity. The software helps to establish temperature-dependent relationships between the transitions from one stage to another during the life history of an insect [29]. Statistical criteria, such as the Akaike’s information criterion (AIC) [42], which are inbuilt in ILCYM, were used to select the mathematical expression for each life stage of the pest with the best fit.

#### Development time and its variation

The values of the development rates of DBM obtained from laboratory experiments at different temperatures were normalized and fitted to density distribution functions. The cumulative frequencies of developmental times of each life stage and temperatures were plotted against normalized developmental times. Logit distribution curve [25,29] was considered as the best fit function for the egg, larva, pupa and adult male, whereas a complementary log—log (CLL) distribution curve [25] was considered as the best fit function for the adult female.

#### Development rate

Both linear and non-linear models were evaluated for fitting the development rates for each immature stage of the pest. The low temperature thresholds and thermal constants were derived from the fitted linear regression model,

r(T)=a+bT

where, *r(T)* is the development rate at temperature T, *a* is the intercept and *b* the slope of the equation. The values of development rates obtained by inversing the median development times (development rate = 1/ development time) were fitted best to Logan 1 [43] model. This function helps to represent and describe the temperature-dependent development rates of egg and larva. Hilbert and Logan model [44] proved to be the best fit for describing development rate of DBM pupa.

#### Mortality of immature stages

Mortality of eggs was best described by the Weibull function [45] whereas a second order polynomial expression [46] offered the best fit for mortality of the larvae and pupae, respectively.

#### Adult life span and aging

Tanigoshi model [43] fitted best to describe the relationship between the age of adult females and temperature. The modified Hilbert and Logan model [44] allowed a good representation of the senescence of adult males. Female longevity was measured and the following parameters calculated:

Age-specific survival (l_x_) of females at 10, 15, 20, 25 and 30°C.

Expected remaining life span (*Ex*) of females: $Ex=\sum_{y=x}\frac{ly+ly+1}{2}/lx$ [47]

#### Reproduction

A simple Gaussian function [48] was considered as the best fitted model in expressing the effects of temperature on fecundity. On the other hand, the relative oviposition frequency, which shows proportion of total lifetime reproductive potential that elapses during each time period, was evaluated in relation to the normalized age of females at a given temperature. The cumulative oviposition rate was plotted against the normalized age expressed as a ratio of age in days over the mean survival time. The Gamma function [49] was used to fit the experimental datasets and further help to estimate parameters such as:

- Age-specific fecundity (m_x_) (=females born/female) by multiplying the mean number of eggs by ratio of females to total population [50].
- Reproductive value (V_x_) of females: $V_{x}=\frac{\sum_{y=x}\left(e^{r_{m}.y}.\;\;l_{y}\;.\;m_{y}\right)}{l_{x}\;.\;\;e^{-r_{m}\;.\;x}}$ [51]

#### Population growth parameters

Once the phenology model was developed, the “validation and simulation” module imported the models from “model builder” and applied rate summation and cohort updating approaches to estimate the population growth parameters of DBM. The estimates were generated from stochastic simulations under constant temperatures with 10 repetitions. An initial population of 100 individuals and the estimated population growth parameters were plotted against the respective temperatures and fitted to cubic equations [52].

#### Model validation

The validation tool in ILCYM tests the ability of developed phenology models to reproduce similar physiological behavior of the insect under fluctuating temperature conditions [41]. A complete life table experiment was conducted outdoors at *icipe* (1619 m above sea level; latitude S 01.22051°; longitude E 036.89563°) following the same experimental procedures used for constant temperatures in the laboratory, from 3^rd^ November– 30^th^ December 2014. A total of 100 individual insects were used. Validation was conducted by using a stochastic simulation, in which results obtained from fluctuating temperatures were compared with phenology model simulation outputs. In addition, the effects of temperature on development time, adult life span, fecundity and population growth parameters obtained from the phenology model were individually compared to published information using the generalized linear modeling (GLM) in R [53] with means separated by Tukey HSD (P < 0.001).

### Climate data used in spatial analysis

The mean minimum and maximum temperature information collected from individual data loggers in each farm in the year 2013 were considered as current climatic conditions. Climate data to represent future climatic conditions (2055) were obtained from the regional climate models (RCMs) at 30” (1km) spatial resolution documented in Platts et al [36]. This is a downscaled, bias-corrected and open source spatial database accessible at AFRICLIM (http://www.york.ac.uk/environment/research/kite/resources/) [37]. To establish the difference between the current and future climates, several steps were involved. The available downscaled values of future temperature (mean daily minimum and maximum) in raster format were loaded and opened in Quantum Geographic Information System software (QGIS) [54]. Every farm was presented with 24 files, in which 12 were the mean daily minimum temperatures covering a month (from January to December) and the remaining 12 were the mean daily maximum temperatures for the same period.

### Spatial analysis of distribution and abundance of DBM along the altitude and over time

The information on field geographical coordinates—altitude and temperature data—was arranged into a spatial framework and linked to the compiled phenology model, to estimate the life table parameters of DBM [30]. Three risk indices, namely establishment risk index (*ERI*), generation index (*GI*) and activity index (*AI*), were produced from the obtained population growth parameters. The *ERI* identifies areas with a favorable climate for survivorship and establishment of the pest, and it is estimated based on a daily time scale by the following expression:

$$ERI=\frac{\sum_{1}^{i=365}I_{i}}{I_{I}}*net-reproduction$$

where, *I*_*i*_ is the interval of day *i* (with *i* = 1, 2, 3,…, 365) and the total number of intervals, *I*_*I*_, is 365.

The index is 1 when all the immature stages of DBM survive throughout the year at varied proportions, with *ERI*<1 characterizing areas in which survival and establishment of the population is restricted to certain periods in the year.

The *GI* estimates the mean number of generations the pest can produce per annum, calculated by averaging sum of the estimated generation lengths in each Julian day as shown in the formula:

$$GI=\frac{\sum_{X=1}^{365}365/Tx}{365}$$

where, *Tx* is the predicted generation length in days at Julian day *x* (*x* = 1, 2, 3,…, 365). When temperature is rising, it theoretically implies more number of generations per year. However, in practice, extreme temperatures reduce fecundity and increase mortality, disrupting the finite rate of natural increase (*λ*).

The third parameter, *AI*, is related to annual finite rate of natural increase of the DBM population, taking into account the whole life history of the pest. The index is calculated by taking a log of products of the estimated finite rates of natural increase for each Julian day as shown below:

$$AI={\text{log}}_{10}\prod_{x=1}^{365}\lambda x$$

where, *λx* is the finite rate of increase at Julian day *x* (*x* = 1, 2, 3,…, 365). Every increase of *AI* by 1 implies a 10-fold increase of the pest population [55].

Using the index interpolator (a sub-module of ILCYM), the compiled DBM phenology, the Digital Elevation Model (DEM) defined by geo-referenced altitudinal data obtained from the Shuttle Radar Topography Mission (SRTM), and the temperature data in text files, were imputed into ILCYM and the pest risk indices for each transect generated in form of American Standard Code for Information Interchange (ASCII) formats [56]. Obtained results were transferred into QGIS for analysis, visualization and interpretation.

## Results

### Temperature-dependent life tables for diamondback moth and phenology model

#### Development time

Mean development times of the immature stages varied significantly with temperature (eggs: χ^2^ = 321.6, df = 5, 930, P < 0.001; larvae: χ^2^ = 178.63, df = 5, 741, P < 0.001, pupa: χ^2^ = 254.27, df = 5, 511, P < 0.001). The mean development times (where accumulated development frequency = 50%) decreased at all life stages with increasing temperatures. The mean developmental time of DBM egg varied from 20.6 days at 10°C to 2.5 days at 35°C. The mean developmental time of DBM larva was approximately 10 times higher at 10°C than at 35°C. The same trend was observed in the mean developmental times of pupae (Table 1).

**Table 1:** Mean development time of DBM life stages at different constant temperatures.

Temperature (°C)	Mean development time (No. of days ± SE)		
	Egg (n = 936)*	Larva (n = 747)	Pupa (n = 517)
**10**	20.64 ± 0.20a	25.55 ± 1.65a	27.45 ± 1.04a
**15**	8.68 ± 0.27b	19.30 ± 0.62b	14.84 ± 0.35b
**20**	5.40 ± 0.10c	11.78 ± 0.20c	7.51 ± 0.09c
**25**	3.47 ± 0.07de	7.89 ± 0.21d	4.84 ± 0.10d
**30**	3.43 ± 0.09e	5.51 ± 0.12e	3.79 ± 0.0.07ef
**35**	2.55 ± 0.051f	2.71 ± 0.24f	2.86 ± 0.20f

Mean values within a column followed by a different letter differ significantly at P<0.05, Poisson and Negative binomial GLM (Tukey test).

*n = number of immature stages observed

#### Development rate

**Fig 1:**
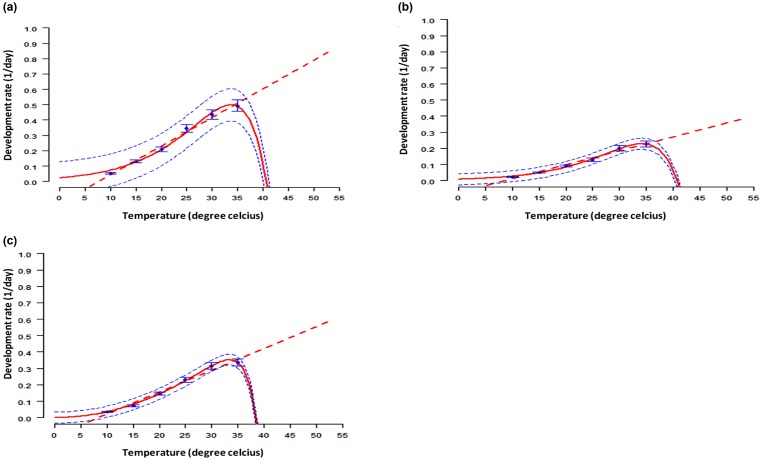
Development rates of immature stages of DBM: Egg (a), larva (b) and pupa (c). Blue dots are the observed means ± SE, the solid red line represents the selected model output, while the dotted blue lines represent the upper and lower 95% confidence limits. Bars represent standard deviations.

The estimated lower development threshold temperatures were 3.76°C for egg (F = 273.1, df = 1, 4 and P < 0.001), 4.79°C for larva (F = 355.5, df = 1, 4 and P < 0.001) and 4.21°C for pupa (F = 305.7, df = 1, 4 and P < 0.001). The thermal constants expressed in degree days (DD = 1/slope) to represent the amount of energy needed to complete development for eggs, larvae and pupae were estimated at 22.4, 58.5 and 37.1, DD respectively. Optimal values of temperatures reached 32.5 (eggs), 33.5 (larvae) and 33°C (pupae), respectively (Fig 1). Beyond these values, the model predicted a sharp decline in the development rates at all immature stages of DBM. The upper threshold limit for development of eggs larvae and pupae was observed at 40.6, 40.7 and 38°C, respectively.

#### Mortality of immature life stages

**Fig 2:**
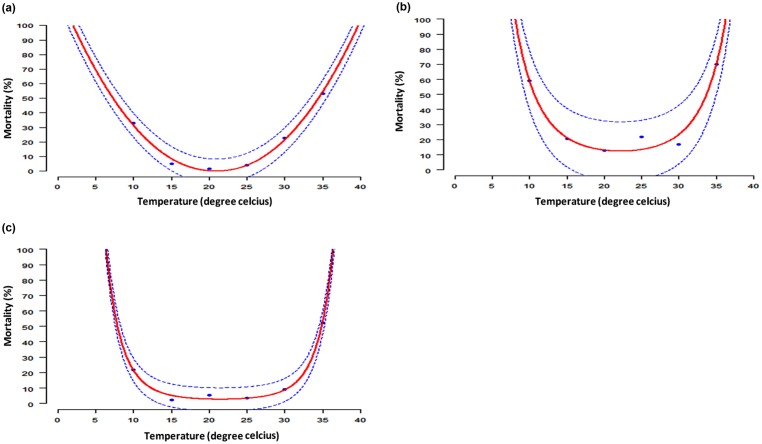
Temperature-dependent mortalities of immature life stages of DBM: Egg (a), larva (b) and pupa (c). Blue dots are the observed means, the solid red line represents the selected model output, while dotted blue lines represent the upper and lower 95% confidence intervals of selected models.

Mortality of immature life stages of DBM was best described by Weibull function for eggs and a second order polynomial for larvae and pupae. The models predicted 20°C as the most favorable temperature level for the survival of eggs, larvae and pupae (Fig 2). The rates of mortality of immature life stages of DBM increased at extreme temperatures.

#### Adult lifespan and reproduction

The adult lifespan and fecundity differed significantly at extreme temperatures (lifespan: male; χ^2^ = 51.39, df = 5, 240, P < 0.001; female: χ^2^ = 37.22, df = 5, 223, P < 0.001; fecundity: χ^2^ = 318.12, df = 4, 215, P < 0.001). The lifespan of DBM females reached its peak of 58.46 days at 10°C whereas the lifespan of DBM males reached its peak at 44.09 (10°C) and 44.73 days (15°C). Overtime, the life span of adult DBM males was shorter than for females at 10°C, but females aged faster in subsequent temperatures (Table 2).

**Table 2:** Lifespan and fecundity of DBM at constant temperatures.

Temperature (°C)	Longevity (in days)		Fecundity [No. eggs/female]
	Male (n = 246)*	Female (n = 229)	(n = 220)
**10**	44.73 ± 3.82a	58.46 ± 3.89a	26.83 ± 10.09a
**15**	44.09 ± 2.68a	31.89 ± 1.84b	155.64 ± 18.72bde
**20**	31.97 ± 1.21b	22.61 ± 0.84c	265.21 ± 13.24cd
**25**	21.34 ± 1.6cd	14.04 ± 0.91de	197.83 ± 14.39d
**30**	20.17 ± 1.03d	13.90 ± 0.79e	101.54 ± 11.80e
**35**	7.33 ± 2.03e	6.43 ± 0.78f	-

Mean values within a column followed by different letter differ significantly at P<0.05, Poisson and Negative binomial GLM (Tukey test)

* n = number of adults observed

**Fig 3:**
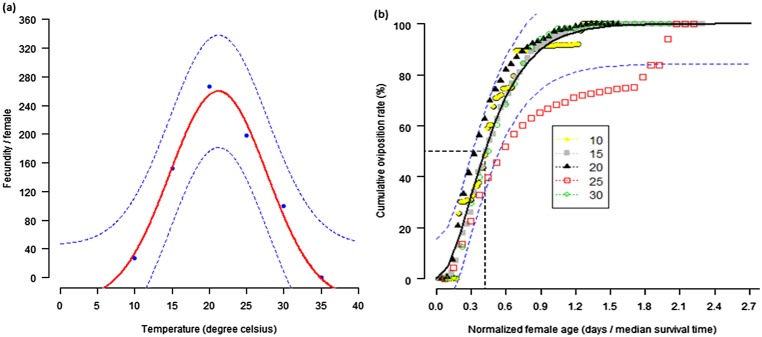
Temperature-dependent total egg production (a) and age-related cumulative proportion of egg production (b). Age of the females at 50% oviposition is indicated. Dots represent data points. The upper and lower 95% confidence intervals of the model are indicated.

The temperature-dependent fecundity of DBM was described by a simple Gaussian function (P = 0.0191; df = 3, 2; F = 51.5897). The model predicted a value near 20°C to be the favorable temperature for DBM females to oviposit. Under this condition, a female produced an average of 265.2 eggs. At a constant temperature of 35°C, only a few adult females emerged from the pupae, but all failed to oviposit. At low and high temperatures, fecundity reduced considerably (Fig 3a). The relationship between cumulative oviposition rate and female age was described by the gamma function (P < 0.001; df = 1, 246; F = 3780.426) (Fig 3b).

Fifty (50) percent of the eggs were laid on the first 44.1, 31.9, 36.4, 13.8 and 11.5% of the adult lifetime, at 10, 15, 20, 25 and 30°C, respectively. A summary of the DBM life table is presented in Table 3.

**Table 3:** Summary of the life table of DBM at different constant temperatures.

Day	Age specific survival (l_x_)	Age specific fecundity (m_x_)	Reproductive value (V_x_)	Expected life expectancy (E_x_)
10°C (n = 24)*				
**30**	0.96	0.1	0.2	5
**34**	0.86	0.1	0.1	4.2
**38**	0.84	1.5	2.6	3.7
**45**	0.75	0	0	3.3
**58**	0.83	0	0.1	3
**66**	0.9	0	0	2.4
**15°C (n = 46)**				
**11**	0.81	2	3.9	3.6
**22**	0.89	2.8	4.9	3.2
**28**	0.74	1	1.6	2.5
**31**	0.57	0.1	0.2	2
**35**	0.38	0	0	1.7
**42**	0.8	0.4	0.4	1
**20°C (n = 57)**				
**7**	0.95	10.3	20.5	3.6
**12**	0.92	5.9	11.4	3
**16**	0.73	2.3	4.3	2.1
**21**	0.36	2.6	3.9	2.3
**25**	0.46	0.4	0.5	1
**25°C (n = 46)**				
**4**	0.89	7.6	14.9	3
**8**	0.67	2.4	4.4	2.2
**12**	0.5	1.8	2.8	1.8
**16**	0.54	1.4	1.8	1.7
**20**	0.29	0	0	1.3
**30°C (n = 47)**				
**3**	0.98	9.7	19	3.3
**7**	0.76	5.2	10	2.4
**11**	0.47	2.5	4.3	1.8
**15**	0.56	0	0	1.7
**19**	0.22	0	0	1.2

* n = number of adults observed. At 35°C, no adult survived

#### Population growth parameters of DBM

**Fig 4:**
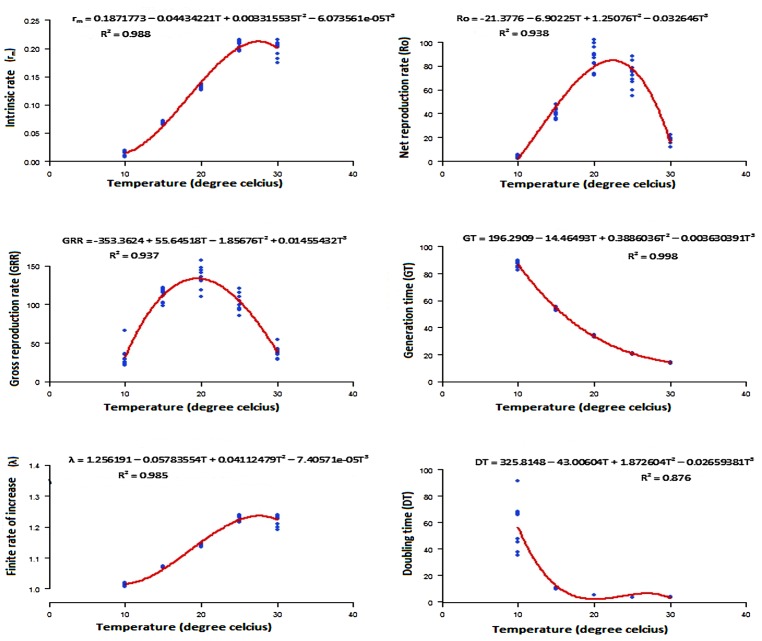
Population growth parameters of DBM estimated over a range of five constant temperatures. r_m_, intrinsic rate of natural increase; Ro, net reproduction rate; GRR, gross reproduction rate; GT, mean generation time; λ, finite rate of increase; and DT, doubling time.

The intrinsic rate of natural increase of the population reached 0.21 at 25°C, suggesting this is the optimal temperature. The pest obtained a maximum net reproduction rate of 87.38 female offpring/female/generation at 20°C. Under the same conditions of temperature, the total number of offspring reached 136.12 individuals/female/generation. The time lags between the same life stages from one generation to the next reduced with increased temperature. Though the values did not differ substantially with temperature (χ^2^ = 0.00268, df = 4, 45, P = 0.9889), the finite rate of population increased steadily from 10 to 25°C before starting to decline. An increase of temperature values from 10 to 25°C reduced the time period to double the population (Fig 4; Table 4).

**Table 4:** Estimated population growth parameters of DBM at different constant temperatures.

Temperature (°C)	Population growth parameters					
	r_m_	Ro	GRR	GT	λ	Dt
	(n = 50)	(n = 50)	(n = 50)	(n = 50)	(n = 50)	(n = 50)
**10**	0.01 ± 0.001a	3.31 ± 0.36a	32.44 ± 4.15a	86.92 ± 0.75a	1.01 ± 0.001a	56.96 ± 5.57a
**15**	0.07 ± 0.001a	41.22 ± 1.25b	110.77 ± 2.91bcd	54.31 ± 0.29b	1.07± 0.001a	10.14 ± 0.11b
**20**	0.13 ± 0.001a	87.38 ± 3.19c	136.12 ± 4.41c	33.67 ± 0.18c	1.14 ± 0.001a	5.23 ± 0.05cde
**25**	0.21 ± 0.002a	72.59 ± 3.28d	102.8 ± 3.37d	20.63 ± 0.09d	1.23 ± 0.003a	3.35 ± 0.04de
**30**	0.20 ± 0.004a	17.08 ± 1.03e	38.35 ± 2.31a	14.11 ± 0.08e	1.22 ± 0.005a	3.48 ± 0.08e

Mean values within a column followed by different letters differ significantly at P<0.05, Poisson and Negative binomial GLM (Tukey test). r_m_, intrinsic rate of natural increase; R_o_, net reproduction rate; GRR, gross reproduction rate; GT, mean generation time; λ, finite rate of increase; Dt, doubling time (days). Adult female DBM failed to reproduce at 35°C.

#### Phenology model evaluation

The minimum and maximum temperatures recorded ranged between 13.1–18.6 and 22.6–38.6°C, respectively. The mean temperature was 21.8°C. The simulated values of intrinsic rate of natural increase (r_m_), finite rate of population increase (λ), doubling time (Dt), and mortality were closely similar to the observed values (Table 5), signifying the strength of the developed phenology models in estimating demographic parameters.

**Table 5:** Validation of the developed phenology model through comparison of observed and simulated population growth parameters of DBM life stages.

								Euclidian distance	
**Population growth parameters**				**Phenology model**				Egg	41.83
**Parameter**	**Simulated**	**Observed**	**P-value**	**Mortality**	**Simulated**	**Observed**	**P-value**	Larva	75.48
**r**_**m**_	0.16(0.01)	0.16	0.31	Egg	0.12(0.05)	0.12	0.5712	Pupa	103.09
**λ**	1.18(0.01)	1.17	0.3054	Larva	0.17(0.08)	0.18	0.2611	Female	41.29
**Dt**	4.28(0.32)	4.34	0.3734	Pupa	0.55(0.13)	0.54	0.8497	Male	42.29

Standard errors are enclosed in brackets.

n = 100.

### Temperature changes between the current and future scenarios

It was estimated that changes in the maximum temperatures between the current and future will be substantial (1.6°C) in the low zone of Taita hills and will reduce considerably towards farms located at the higher altitudes in the transect (Table 6). The changes will be comparatively lower and uniform along the Mt. Kilimanjaro transect.

**Table 6:** Changes between the current (2013) and future (2055) maximum and minimum temperatures on selected farms along Mt. Kilimanjaro transect and the Taita hills transect. All current, future and their differences in temperatures were recorded in degrees centigrade (°C) and altitude in metres above sea level (masl).

	Mt. Kilimanjaro					Taita hills				
**Maximum temperatures**										
**Zone**	**Farm**	**Altitude**	**Current**	**Future**	**Difference**	**Farm**	**Altitude**	**Current**	**Future**	**Difference**
**Low**	Kisange B	716	34.3	34.8	0.4	Majengo	830	33.8	35.4	1.6
**Medium**	Kirua	1513	28.3	28.6	0.3	Msangalinyi	1461	27.7	28	0.3
**High**	Marua A	1692	26.8	27.2	0.4	Kishamba	1765	25.9	25.9	0
**Minimum temperatures**										
**Low**	Kisange B	716	20	20.9	0.9	Majengo	830	17.3	19.2	1.9
**Medium**	Kirua	1513	15	15.7	0.7	Msangalinyi	1461	13.1	15.8	2.7
**High**	Marua A	1692	13.3	14.5	1.2	Kishamba	1765	13.1	13.8	0.7

### Spatial changes in distribution and abundance of DBM population

#### Current scenario along the altitudinal transect

**Fig 5:**
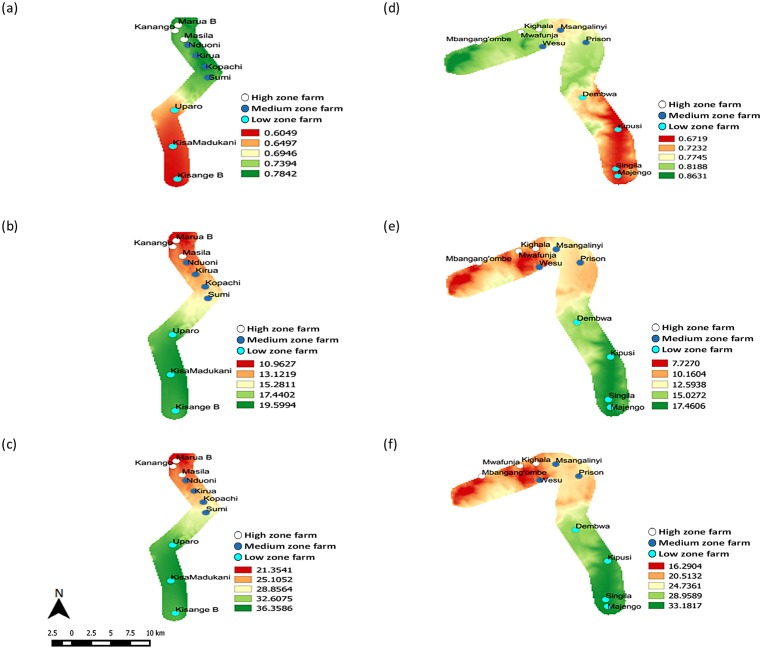
Changes in the establishment, abundance and population growth rates of DBM along altitudinal gradients of Mt. Kilimanjaro and Taita hills. Establishment risk indices (*ERI*) of Mt. Kilimanjaro (a) and Taita hills (d); Generation indices (*GI*) of Mt. Kilimanjaro (b) and Taita hills (e); and Activity indices (*AI*) of Mt. Kilimanjaro (c) and Taita hills (f) *KisangeB = Kisangesangeni B, KisaMadukani = Kisangesangeni Madukani.

The current suitability of habitat for the survivorship and establishment of DBM increased gradually from 700 masl in the low zone (*ERI* = 0.6049) to 1690 masl in the high zone (*ERI* = 0.7842) in Mt. Kilimanjaro (Fig 5a), reflecting the observed distribution of the pest in the transect. In contrast, the highest number of new generations of DBM added were in the low zone (*GI* = 19.5994), with the number reducing steadily with increasing altitudes in the transect, settling to approximately 11 per annum in the high zone (Fig 5b). The temperature-dependent population increase is shown to decrease consistently from a range of approximately 363.6-fold at the bottom of the low zone (*AI* = 36.3586) to approximately 213.5-fold at the top of the high zone (*AI* = 21.3541) (Fig 5c).

Suitability of habitats based on temperature increased with increasing altitude in Taita hills. If you consider two farms, one at Majengo (830 masl) and the other at Dembwa (1107 masl), little variation in the level of suitability of habitat is noticed in the low zone (0.6719 ≤ *ERI* ≤ 0.7232) (Fig 5d). From medium towards high zone, the farms become increasingly suitable, with the likelihood of pest establishment ranging between 0.7745 and 0.8631. However, it was observed that the pest produces increasingly fewer new generations with rising altitude, reducing from 17.5 generations (*GI* = 17.4606) in the low zone to 7.7 generations (*GI* = 7.727) in the high zone (Fig 5e). Population growth is roughly halved from a 331.8-fold increase in the low zone (*AI* = 33.1817) to about 162.9-fold increase (*AI* = 16.2904) in the high zone (Fig 5f).

#### Changes between the current (2013) and future climate change scenarios (2055)

**Fig 6:**
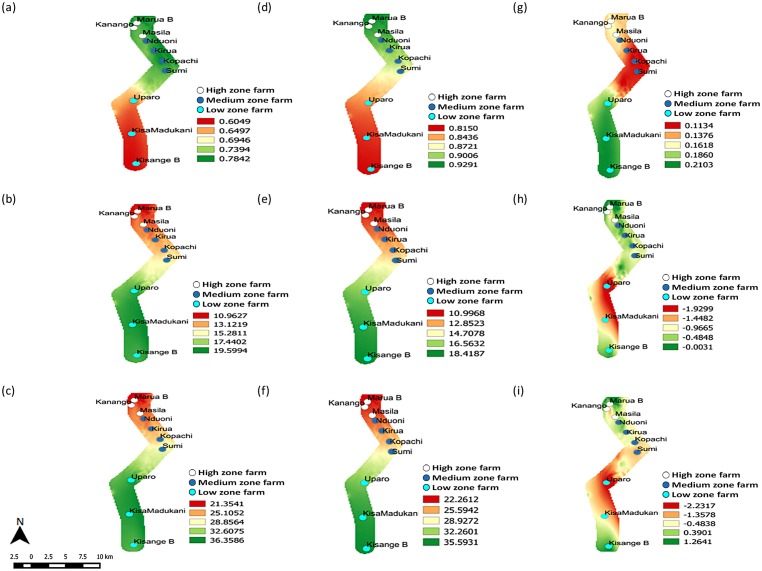
Changed establishment, abundance and population growth rates across climate change scenarios of Mt. Kilimanjaro. Current 2013 distribution and abundance of DBM: (a) *ERI*, (b) *GI* and (c) *AI*; future 2055 distribution and abundance of DBM: (d) *ERI*, (e) *GI* and (f) *AI*. Absolute change in distribution and abundance between current and future scenarios: (g) *ERI*, (h) *GI* and (i) *AI*. *ERI* = Establishment Risk Index, *GI* = Generation Index and *AI* = Activity Index. *KisangeB = Kisangesangeni B, KisaMadukani = Kisangesangeni Madukani.

Most changes in potential distribution between the current (2013) and future (2055) scenarios in Mt. Kilimanjaro will happen in the low zone (Fig 6g) whose establishment risk index ranges from 0.1860 to 0.2103. In between the two periods, the capacity of DBM to produce new generations will reduce, resulting into a loss of approximately two generations (*GI* = -1.9299) in the low zone (Fig 6h). This decline in number of generations will be reflected in the overall population that will be characterized by a decline of the growth rate from the Kisangesangeni Madukani farm upwards to 22.3-fold in the low zone (Fig 6i).

The model predicts that most areas of the medium zone of Mt. Kilimanjaro will be least favorable for survival and establishment of DBM (*ERI* = 0.1134) along the gradient (Fig 6g), leading to a reduction of the mean number of new generations added from 0.95 to 0.42 (Fig 6h). Changes in finite rate of population increase will be associated with the declining growth rate (-1.3578 ≤ *AI* ≤ -0.4838); however, these will not be large enough to warrant significant change in the population growth rate in the zone (Fig 6i).

Population changes in the high zone of Mt. Kilimanjaro are associated with the increase of favorable temperature conditions for the establishment and distribution of the pest (increase of establishment risk index from 0.1134 to 0.1376 (Fig 6g)), which will further influence the number of generations and population growth. The increase in level of suitability will probably allow more DBM to shift into the zone; and thereby, add several new generations in the population (Fig 6h). The added new generations are likely to lead into a net increase of the population growth rate from approximately 3.9 to 12.6-fold.

**Fig 7:**
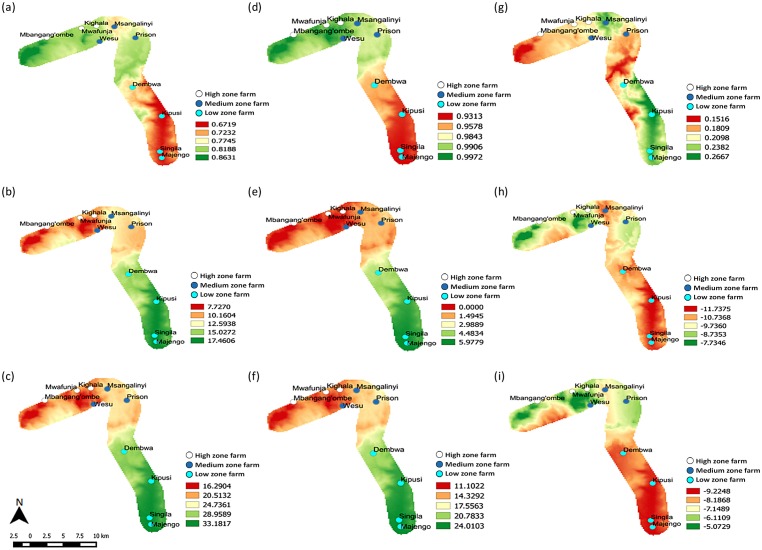
Altered establishment, abundance and population growth rates across climate change scenarios of Taita hills. Current 2013 distribution and abundance of DBM: (a) *ERI*, (b) *GI* and (c) *AI*; future 2055 distribution and abundance of DBM: (d) *ERI*, (e) *GI* and (f) *AI*. Absolute change in distribution and abundance between current and future scenarios: (g) *ERI*, (h) *GI* and (i) *AI*. *ERI* = Establishment Risk Index, *GI* = Generation Index and *AI* = Activity Index.

The model also predicts that the changes in favorable habitats between current and future temperature conditions in Taita hills will be substantial in the low zone farms (0.2098 ≤ *ERI* ≤ 0.2667) (Fig 7g). By 2055, the number of generations produced per annum will decline gradually, with a loss of approximately 12 generations alone in the lowest-lying farm in the transect, Majengo (Fig 7h), leading to a significant reduction of DBM population growth in the zone. The farm is predicted to lose about 92.2 times of its DBM population between these two periods (Fig 7i).

A wider range of the establishment risk index in the medium zone of Taita hills spanning from 0.1809 to 0.2667 (Fig 7g) can only suggest more suitable conditions and extensive favorable temperatures between current and future conditions. Nonetheless, the predicted changes seem unfavorable to DBM population, because, as the expected added number of generations reduces to approximately 8.74 and 10.74 generations, this will slow down the growth rate by 7 to 8-fold (Fig 7i), which will also translate to a reduction in the damage potential in the zone.

In the high zone of Taita hills, the environmental suitability for survivorship of DBM is expected to change little, with such changes becoming even smaller upwards in the zone (Fig 7g). In this zone, therefore, the pest will witness little variation on its annual number of new generations; which is translated into maintaining a more stabilized population compared to the other zones (Fig 7h). Potentially, the overall impact will be an increase of the population growth rate (Fig 7i).

## Discussion

Several bioclimatic models have been developed for determining temperature-dependent population dynamics, abundance and distribution of insect species in the field. Often these models are developed at broad geographic scale, mostly continental or global scale. Utility of such broad geographic scale prediction with scarce inclusion of information on the pest biology is often limited for improving or adapting management strategies in response to climate change at a local scale. In the eastern Afromontane, cruciferous vegetables such as kale and cabbage are widely cultivated in diverse agroecologies spread along the altitudinal gradient, but DBM is a key constraint to productivity of these vegetables [33,57]. The growth and development of DBM are significantly influenced by the prevailing weather variables, especially temperature and rainfall, in the field [58]. Understanding the effects of changing climate on DBM biology and damage to crops is critical for adapting and implementing management strategies in the region. However, previous efforts to predict distribution of DBM in relation to climatic variables using approaches such as CLIMEX [19] have been employed on a global scale and were entrenched on limited information on the pest biology. Regional prediction models, as reported for abundance of DBM in China using DYMEX software [59], are also not robust enough to properly analyze age-structured pest populations. Temperature-dependent life tables provide reliable information for understanding the life history, behavioral response and population dynamics of key insect pests such as DBM, under the widely changing temperature conditions in a local scale [31]. Furthermore, such information is useful for predicting potential impacts of climate change on the population dynamics through estimations of the lower and upper threshold of temperatures during the species life history [28].

We used process-based phenology models to understand the temperature-dependent population growth potential of DBM. Our results indicated that growth and reproduction of DBM could only be sustained between the minimum and maximum temperature thresholds of 3.76°C for eggs and 40.7°C for larvae, respectively. Although the development rate increased with temperature, our findings show that females failed to oviposit at temperatures beyond the maximum threshold of 30°C. Maximum number of eggs and female offspring were only produced at 20°C, suggesting the temperature range of 20–25°C is the optimum for high intrinsic rate of natural increase and maximum net reproduction rate. These results agree with previous reports of Bahar et al [11] who found that 4°C was the lowest temperature threshold for eggs and that no larvae survived beyond 38°C. Marchioro and Foerster et al [26] also reported maximum oviposition by DBM at 20°C, with the highest intrinsic rate of natural increase occurring between 20 and 25°C. Further, 50% of the total number of eggs at 20°C were laid in 36.4% of the female’s life time. Chelliah and Srinivasan [60] point out that DBM is physiologically programmed to lay the maximum number of eggs in the early days following eclosion.

Often, DBM outbreaks in the tropics occur during the hot dry season in the low altitudes soon after a wet season [61] or with ample irrigation resources [62] when there are abundant food supplies. The growth of DBM outside the optimum temperature range declined as evidenced by high egg mortality towards both the lower and upper temperature extremes (10°C and 30°C). A long period of exposure to temperature beyond 32.5°C impaired viability of the eggs, resulting in decline of field populations [26]. At temperatures, lower than optimum, longer development times resulted in fewer numbers of generations per year in the field. Exposure to freezing temperatures for less than two months could lead to hibernation of DBM [63]; however, such weather is uncommon to crucifer cultivation in the tropics. Similarly, with increasing temperatures above the optimum, shorter development times could lead to more generations per year in the field. However, this is not always the case in nature, because the relatively long periods of exposure to high temperatures interfere with food conversion-enzymes [64] and deprive the pupae and adults the possibility of gaining the normal weight for proper growth and differentiation [11,64].

Although the experiments were conducted at constant temperatures, in nature, temperatures fluctuate both due to diurnal variations and microclimate. Hence, when periodically stressed from the very hot or cold weather, insects limit their movements, only to resume normal activity when favorable conditions resume [65–66]. Liu et al [67] reported that eggs incubated for up to 36 hours at a constant temperature of 38°C could still develop when transferred to 28°C. Thus, to test reliability of the models developed under constant temperatures, it may be reasonable to compare species performance under a close range of fluctuating temperatures. However, only a few studies have investigated the effect of fluctuating temperatures on mortality and population growth parameters of DBM [66,68–69]. Under higher temperature fluctuations of 25 ± 10°C, 57% of the eggs of DBM died at 37°C [67], which is comparable to 53.3% mortality observed in this study. Similarly, mortality rate of pupae (5.6%) observed at 20°C in the present study is comparable to mortality rate of 8.5% under fluctuating temperature (15–27°C, mean: 22°C) reported previously [66]. Our adopted models predicted the higher survival of pest population under moderate temperatures [68], which supports our observations on suitability of medium and high zones for DBM population buildup.

To understand the changes in population dynamics of DBM with graded changes in the weather variables along the altitudinal transects, we ran the developed phenology models with field collected temperature datasets from Mt. Kilimanjaro and Taita hills. Both in Mt. Kilimanjaro and Taita hills, suitability of habitats for survivorship and establishment increased from the low-altitude (*ERI* = 0.6049–0.6719) to the high-altitude zones (*ERI* = 0.7842–0.8631). However, with number of generations per year declining from 17–19 in the low altitude zones to 8–11 in the high zones, temperature-dependent population growth rate is nearly double in the low zones compared to the high zones. In addition, the shorter generation length in the low altitude zone implies a rapid increase in populations [31]. However, our findings indicate that this population growth trend will only hold if the pest population is not exposed for long periods at or above 35°C [15], which would otherwise compromise egg production, pupation and adult size [70].

Several studies have suggested that warming gets more rapid at higher elevations in the tropics [35] and particularly so, when the altitudinal gradient is constant. Based on the current (2013) and future (2055) temperatures on selected farms along each transect, significant increases in mean minimum temperatures could be expected in Mt. Kilimanjaro (+0.7–1.2°C) and Taita hills (+0.7–2.7°C), respectively. In the low altitude zone of Taita hills, increase of mean maximum temperature up to 1.6°C could also be expected. Change of mountain temperatures is also subject to the extreme local variability in the topography, slope, aspect, tree cover and exposure [35,71]. It is possible the well-managed “Chagga homegardens”, a multi-storeyed agroforestry cropping system based on banana and coffee, providing shading effects in the upward slope of Mt Kilimanjaro [39,72], enhanced resilience of the crucifer cropping systems to harsh climates by providing the favorable microclimate [73–77]. Taita hills, which has lost approximately 99% of its forest cover in the past few centuries [38] is likely to experience more warmer climates [37] compared to Mt. Kilimanjaro where nearly one third of the forest cover has been lost in the last 70 years [78]. The falling coffee prices in the world market, rising production costs and changing climate [76,79], to mention a few, have compelled some farmers in Mt. Kilimanjaro to substitute coffee with other food and cash crops [80]. To this end, the diversity, density and placement of existing shade trees have been changed, leading to significant reduction in the tree component of the Chagga homegardens [76].

Based on predictions of the DBM population risk indices in a future climate change scenario (2055) along the transects, the crucifer farms in the low zone of Mt Kilimanjaro near Kisangesangeni B and farms in higher altitudes of the transect from Kirua to Marua B are likely to face increasing pressures of DBM. The higher change in the population growths expected in the low zones of Mt Kilimanjaro indicates the possible role of Miwaleni springs, which is closest to the Kisangesangeni B farm, in producing the microclimate needed for both host plants and DBM in a region that is otherwise relatively hot. Furthermore, in the mid to high zones of Mt Kilimanjaro, the Chagga homegardens [39] could buffer DBM growth from unfavorable extreme temperatures.

However, significant declines in the *GI* across the Taita hills transect by 8–12 generations and declines in the *AI* from -5.0729 to -9.2248 due to rising temperatures in the future could lead to an overall decline in population of DBM across the transect. As noted in numerous studies [9–10,81], high field temperatures will not necessarily increase population of the pest but might impair the physiology of the pests directly and indirectly through increased killing capacity of its predators. The decline in egg oviposition and hatchability of DBM after 20 and 32.5°C, respectively [26], and the decline in finite rate of increase after 25°C, will inevitably cause a decrease of the DBM population in the low zone. The high zones of Taita hills will undergo significant increase in DBM dynamics with greater potential for survival of all life stages of DBM throughout the year with optimum temperature conditions, resulting in significant yield losses. However, with increasing temperature, the potential for outbreak of other pests such as thrips and aphids [82–83] in the low zones needs to be considered.

In conclusions, the study highlights how detailed temperature-based life analysis of a pest (such as DBM) in the laboratory combined with field-collected and downscaled temperature data could predict the dynamics of the pest at a local scale both under current and future climate change scenarios. In a broader sense, incorporating other factors known to influence DBM dynamics into the model such as weather-influenced reproductive and dispersal behavior of the pest [84–86], host plant diversity [12–13,40], natural enemy dynamics [32,87], relative humidity [88–89] and rainfall [90–91] could enhance the validity of these predictions. A recent study has highlighted the relevance of microclimate in influencing the bioecology of insect species [92]; hence incorporation of fine scale weather data for modeling species distribution can also further enhance the precision of the predictions.
